# Guanidinoacetic Acid Significantly Improves Growth, Antioxidant Capacity, and Nonspecific Immunity for Juvenile *Litopenaeus vannamei*

**DOI:** 10.1155/anu/5538869

**Published:** 2025-04-13

**Authors:** Huaxing Lin, Beiping Tan, Shuyan Chi, Qihui Yang

**Affiliations:** ^1^College of Fisheries, Guangdong Ocean University, Zhanjiang 524088, China; ^2^Guangdong Engineering Technology Research Center of Aquatic Animals Precision Nutrition and High Efficiency Feed, College of Fisheries, Guangdong Ocean University, Zhanjiang 524088, China; ^3^Guangdong Provincial Key Laboratory of Aquatic Animal Disease Control and Healthy Culture, College of Fisheries, Guangdong Ocean University, Zhanjiang 524088, China

**Keywords:** antioxidant capacity, growth, guanidinoacetic acid, *Litopenaeus vannamei*, nonspecific immunity

## Abstract

Guanidinoacetic acid (GAA)—a nutritional additive—is essential for the healthy growth of aquatic animals. The experiment was conducted to examine the effects of dietary GAA on growth, muscle amino acid composition, antioxidative indices, and nonspecific immunity for juvenile *Litopenaeus vannamei*. Total 800 healthy shrimp (initial mean weight = 0.27 ± 0.03 g) were equally distributed into 15 tanks (0.3 m^3^; five groups, and three repeats per group) and fed with diets containing GAA levels (e.g., 0, 0.04%, 0.10%, 0.13%, and 0.16%, named G0, G004, G010, G013, and G016, respectively) for 8 weeks (four times a day). At the end of the trial, shrimps from all replicate groups were weighed, and serum, hepatopancreas, and muscle were collected from three random tails. The weight gain rate (WGR) and specific growth rate (SGR) were significantly higher, and feed conversion rate (FCR) was significantly lower in G010 and G013 groups than in G0 group. No significant effect of GAA on the total amino acids of each treatment was observed. Serum superoxide dismutase (SOD) activity and total antioxidant capacity (T-AOC) were significantly higher, and malondialdehyde (MDA) levels were significantly lower in G010, G013, and G016 groups compared to G0 group. Alkaline phosphatase (AKP), phenoloxidase (PO), lysozyme (LZM), and acid phosphatase (ACP) activities were significantly higher in G010, G013, and G016 groups than in G0 group. The mRNA expressions of immune deficiency (*imd*) and *lzm* genes in G010 and G013 groups were significantly upregulated. Following the challenge with *Vibrio harveyi*, the overall percent mortality of shrimp showed a gradually decreasing trend with the increase of GAA supplementary but was not significantly different from each other. In conclusion, GAA can improve the growth, antioxidant ability, and nonspecific immunity for *L. vannamei*.

## 1. Introduction

*Litopenaeus vannamei*, also known as South American white shrimp, is one of the economically important shrimp species in China, with more than 1,429,832 tones in production by 2023 [1]. Of these, *L. vannamei* accounted for 69.57% of the crustaceans. However, with increased aquaculture intensification, the environmental pollution of aquaculture waters is serious, leading to a significant increase in the frequency of disease in farmed animals, which has seriously inhibited the development of shrimp aquaculture industry [2, 3]. Therefore, it has become a research priority to find safe and efficient feed additives, whereas nutritional modulation is an effective way to improve the resistance to oxidative stress and immunity of aquatic animals [4]. Nutritional feed additives can make up for the lack of feed nutrition and have positive significance on the growth and disease prevention for aquatic animal [5].

Guanidinoacetic acid (GAA), being a vital converter of cellular energy metabolism in the organism (a prerequisite for creatine), can participate in the energy metabolism of tissue cells in the animal body and enhance its antioxidant capacity (AOC) [6]. GAA can be stored for a long time at room temperature and has been approved for use in the PR China (PRC), United States of America (USA) and European Union (EU) [7]. GAA is a precursor substance for the synthesis of creatine, generated by the action of GAA amidinotransferase (GAMT) [8]. Studies have shown that GAA is stable in nature and is therefore commonly used as a source of creatine supplementation [9]. In recent years, relevant studies have shown that supplementing GAA in animal feed can improve animal performance, making it a promising application [10]. As a feed additive, GAA is considered more suitable than creatine because it is cheaper and more chemically stable than creatin [11]. In livestock and poultry, numerous studies have shown that GAA added in diets can improve growth and AOC [12–15]. In aquatic animals, as well as in *Sciaenops ocellatus* [16], *Ctenopharyngodon Idella* [17], *Lctalurus punctatus* [18], *Cyprinus carpiovar Jian* [19], *Oreochromis niloticus* [20, 21], hybrid striped bass [22], and *Sebastes schlegelii* [23], there are reports that GAA improves growth performance, feed conversion rate (FCR), and AOC [5, 19, 24, 25]. In addition, GAA improves immunity and disease resistance in aquatic animals [5, 26, 27].

At present, extremely few studies have been conducted on GAA in aquatic products, of which none have been reported for *L. vannamei*. In order to improve the ability of shrimp to fight diseases and reduce losses to the aquaculture industry due to disease outbreaks, GAA is imperfectly studied as a feed additive to improve immunity and disease resistance in farm animals. This study was carried out with shrimp to fill more data references for the utilization of GAA in aquatic animals. Therefore, this study aimed to evaluate the effects of GAA on growth, AOC, and nonspecific immunity for shrimp, aiming at exploring the appropriate amount for GAA.

## 2. Materials and Methods

### 2.1. Experimental Diets

**Figure 1:**
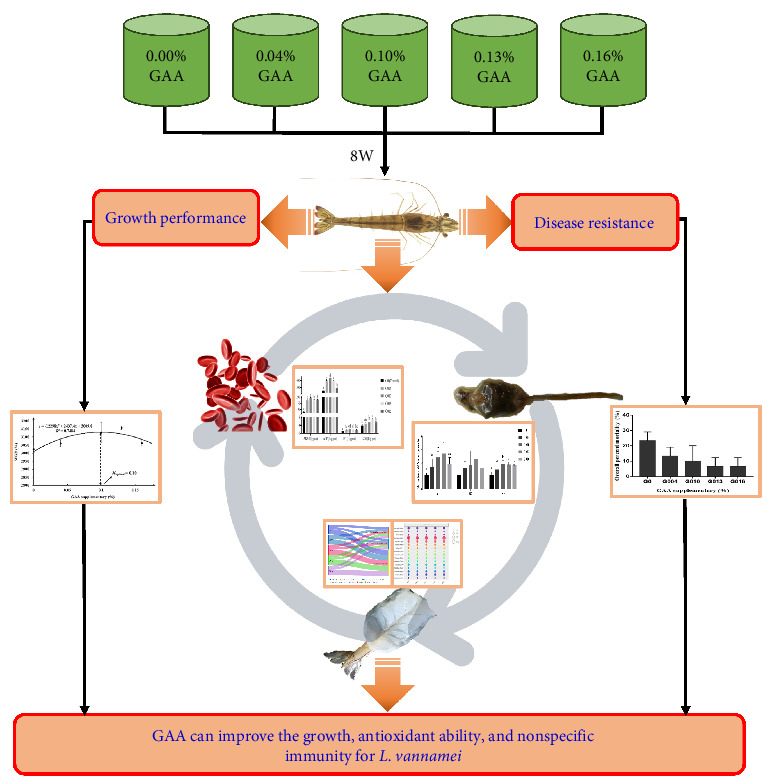
The study map of the effects with GAA for juvenile *L. vannamei*. GAA, guanidinoacetic acid.

Formulations and approximate compositions for the preparation of five isonitrogenous and iso-lipids experimental diets (Table 1). Diets were supplemented with 0%, 0.04%, 0.10%, 0.13%, and 0.16% GAA (purity >99%, provided by Chengdu Boyi Mukang Technology Co., Ltd., Sichuan), and named as G0 (control group), G004, G010, G013, G016, respectively (Figure 1). All the raw materials were shattered using a hammer mill (SF-320, Suzhong Pharmaceutical Machinery Co., Ltd., Jiangsu, China) and sifted through a 178 μm screens mesh. The weight of each raw material is accurately weighed according to the feed formula, and the microingredients (calcium dihydrogen phosphate, VC and premixes, etc.) were added by the step-by-step expansion method, and then, the microingredients and the bulk raw materials are mixed thoroughly with the V-type mixer (South China University of Technology, Guangzhou, China) [28–30]. After adding soybean oil, fish oil, choline chloride, and deionized water, all ingredients were mixed thoroughly in a Hobart-type mixer (M-256, South China University of Technology, Guangzhou, China) to obtain a homogenous diet [31]. Then, each diet was passed through a twin-screw extruder (F-26, South China University of Technology, China) with 1.0 mm and 1.5 mm diameters [32]. Finally, it is dried at room temperature as well as stored at –20°C.

### 2.2. Experimental Shrimp and Feeding Trial

This study followed the recommendations set-out by the Care and Use of Laboratory Animals in China from the Animal Ethical and Welfare Committee of China Experimental Animal Society. The protocol was approved by the Animal Ethical and Welfare Committee of Guangdong Ocean University (Guangdong, China), processing ID GDOU-AEWC-20180063.

Juvenile *L. vannamei* (body length 1 cm) was obtained from Boshang Aquatic Feed Management Department in Xuwen county (Zhanjiang, Guangdong, China). Shrimp were raised in a cement pool (3 m [length] × 3 m [width] × 2 m [height]) with a stocking density of 10,000 shrimp and fed with commercial diet (Zhanjiang Yuehai Feed Co. Ltd., Guangdong, China) until the shrimp reached the experimental specifications (body length 3 cm). After 24 h of starvation, total 600 healthy shrimp (the mean ± standard deviation (SD) weight, 0.27 ± 0.03 g) with regular body shape and uniform size were equally distributed into 15 tanks (five groups and three repeats per group) with 40 shrimp in a completely randomized design. The trial was conducted in an indoor recirculating water system (Marine Biology Research Base, Guangdong Ocean University). Shrimp were reared in 0.3 m^3^ fiber glass tanks with feeding 4 time/day at 7:00, 11:00, 17:00, and 21:00, and feed rates were adjusted depending on changes in growth and water temperature, among other factors [33]. Initially, the feeding rate is 8% per body weight and further adjusted according to the shrimp body weight [34, 35]. Shrimp feeding was observed daily and the daily feeding rate was adjusted. During the experiment, the farmed seawater was sand filtered and continuously aerated, and the water quality indicators were met before use. Water change 1/3–2/3 daily. Among other things, the indicators of water quality must fulfil the following conditions: ammonia nitrogen ≤0.02 mg/L, dissolved oxygen ≥5 mg/L, salinity 28–32 g/L, water temperature 28.4°C–31.2°C, salinity 26–29, pH 7.5–8.0, nitrate, and nitrite <0.10 mg/L. The culture waters were taken every 2 days and tested for ammonia nitrogen and salinity using a portable spectrophotometer (DR1900, HACH, USA). Dissolved oxygen was measured using a portable multiparameter water quality analyzer (HQ2100, Hash, USA).

### 2.3. Sample Collection

At the end of the trial (8 W), shrimps were counted and weighed in each tank to identify shrimps (see Section 2.4) after a 24-h starvation. Before sample collection, shrimp were killed with Tricaine methane sulfonate (MS-222, purity >95%) with a concentration of 2.0 mL/L [36]. Hemolymph samples were collected from the pericardial cavity of four shrimps per tank into 1.5-mL Eppendorf tubes, then left overnight at 4°C, centrifuged for 10 min, and finally, serum was collected and stored (−80°C) for antioxidant index analysis. The hepatopancreas and muscles were taken from the other four shrimps in each tank. Hepatopancreas was collected for the study of innate immune enzyme activities and muscle for the determination of amino acid content. Hepatopancreas from four shrimps were quickly cut into smaller pieces, preserved in RNA later (Ambion, Thermo Fisher Scientific, USA) and then placed in −80°C until analyzed.

### 2.4. Evaluation of Growth Performance

According to the records from the beginning to the end of the experiment, including the initial body weight, daily feed intake, numbers of deaths and actual growth days, etc., weight gain rate (WGR), FCR, specific growth rate (SGR), and survival rate (SR) were calculated [37]. The calculation formula are as follows:

  $$\begin{array}{c} \text{WGR }\left(\%\right)=100\times \left(W_{t}-W_{0}\right)/W_{0}.\text{SGR }\left(\%/\text{d}\right)=100\times \left(\ln\;W_{t}-\ln\;W_{0}\right)/T.\text{FCR}=\text{dry feed intake (g)}/\left(W_{t}-W_{0}\right).\text{SR }\left(\%\right)=100\times N_{t}/N_{0}. \end{array}$$

*Note*: *W*_0_, initial body weight. *W*_t_, final body weight. *T*, actual growth days. *N*_0_, the total number of shrimps at the beginning of the feeding trail. *N*_t_, the total number of shrimps at the end of the feeding trail.

### 2.5. Analysis of Amino Acids in Muscle

The contents of free amino acids in muscle were determined according to national food safety standard (GB 5009.124-2016) [29, 30]. The samples were treated with 6 mol/L hydrochloric acid solution, after freezing and nitrogen filling, hydrolyzed at 165°C for 1 h, and amino acids content were assayed by an automated amino acid analyzer (L-8900, Hitachi High-Technologies Corp., Tokyo, Japan).

### 2.6. Analysis of Antioxidant Index and Immune-Related Enzymes

The malondialdehyde (MDA), superoxide dismutase (SOD), total antioxidant capacity (T-AOC), acid phosphatase (ACP), and alkaline phosphatase (AKP) were assayed using kits from Nanjing Jiancheng Biological Institute [38, 39], whereas phenoloxidase (PO) and lysozyme (LZM) were strictly determined by enzyme-linked immunosorbent assay kits (Shanghai Enzyme-linked Biotechnology Co., Ltd., China).

### 2.7. Analysis of mRNA Expression of Nonspecific Immune-Related Genes

The immune-related genes mRNA expressions in hepatopancreas were evaluated. Total RNA was extracted from shrimp hepatopancreas using the commercial RNA Extraction Kit (TransZol Up Plus RNA Kit, Beijing, China). Then, the total RNA was detected by nucleic acid quantifier SimpliNano. PrimeScript RT-PCR Kit (Accurate Biotechnology (Hunan) Co., Ltd., ChangSha, China) was used to synthesize cDNA. The gene-specific primers are shown in Table 2. All RT-qPCR were performed on a LightCycler480 II System (Roche, Switzerland) by using a SYBR Green Premix *Pro Taq* HS qPCR Kit II (Accurate Biotechnology Hunan Co., Ltd., China). The gene expressions results were calculated using the 2^−ΔΔCT^ method [40].

### 2.8. Challenge Test of *Vibrio harveyi*

The strains of *Vibrio harveyi* used in the experiment were obtained from the Key Laboratory of Pathogenic Biology, Fisheries College, Guangdong Ocean University. The culture of *V. harveyi* was according to Ray et al. [41]. Bacteria were cultured in liquid medium of Luria-Bertani (LB media, Sangon Biotech) for 20 h, then centrifuged for 10 min (4°C, 7000 × *g*). The supernatant was excluded, and the bacteria were cleaned by two washing with sterile phosphate-buffered saline (PBS). Then, the bacteria were serially diluted by PBS to obtain different concentrations, 10^9^, 10^8^, 10^7^, and 10^6^ CFU/mL. Fifty microliters of each concentration of *V. harveyi* were intramuscularly injected into the third abdominal segment of shrimp. Shrimp injected were placed in outdoor tanks (volume, 45 L) containing 35 L of seawater (temperature, 26.5°C; salinity, 31 g/L). The overall percent mortality was recorded for 7 days, and the weighted probability unit method (Bliss method, 1935) was used to determine the median lethal dose (LD_50_, 2.0 × 10^8^ CFU/mL). After the growing experiment, 40 shrimp from each treatment were evenly distributed into four tanks for formal challenge test. Ten shrimp per each experimental group was intramuscularly injected with PBS which was indicated as the negative control. The other three replicate tanks were challenged with 50 μL *V. harveyi* suspension (2.0 × 10^8^ CFU/mL) by intramuscular injection. All conditions during the formal challenge test were the same as those of the pre-experiment. No diets were provided to these animals during the trial. The mortality of shrimp within 7 days was recorded, and the overall percent mortality was calculated [42, 43].

### 2.9. Statistical Analysis

All data were analyzed using SPSS 21.0 software (SPSS Inc, Chicago, IL, USA) for one-way ANOVA. Differences between means were compared using Duncan's multiple comparison test, *p*-values represented whether the difference between the combined diet groups and the control group was significant (*p* < 0.05 considered as significant difference). The figures of genes mRNA expression and the overall percent mortality were plotted by GraphPad prism 8.0 (GraphPad Software Company, San Diego, USA).

## 3. Results

### 3.1. Growth Performance

As GAA increased, WGR, FBW, and SGR all significantly increased before decreasing, and the opposite was true for FCR (Table 3). FBW, WG, and SGR were significantly higher in G010 and G013 groups than in G0 group (*p* = 0.030, 0.030, and 0.040, respectively). The FCR in G013 group was significantly lower than that in G0 group (*p* = 0.014). SR was greater than 95% in all groups but not significantly different (*p* = 0.371).

**Figure 2:**
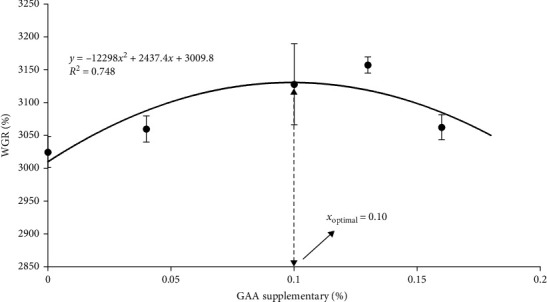
Relationship between GAA supplementary (%) and WGR (%) for juvenile *L. vannamei*. Based on the quadratic regression model of WGR corresponding to GAA supplementary, the optimal supplementary of dietary GAA was 0.10%. GAA, guanidinoacetic acid; WGR, weight gain rate.

There was a quadratic regression relationship between the WGR (*y*) and the level of diet GAA supplementary (*x*) (*y* = − 12,298*x*^2^ + 2437.4*x* + 3009.8, *R*^2^ = 0.7484). The optimal level of supplemental dietary GAA derived from the quadratic regression model was 0.10% (Figure 2).

### 3.2. Amino Acids in Muscle

**Figure 3:**
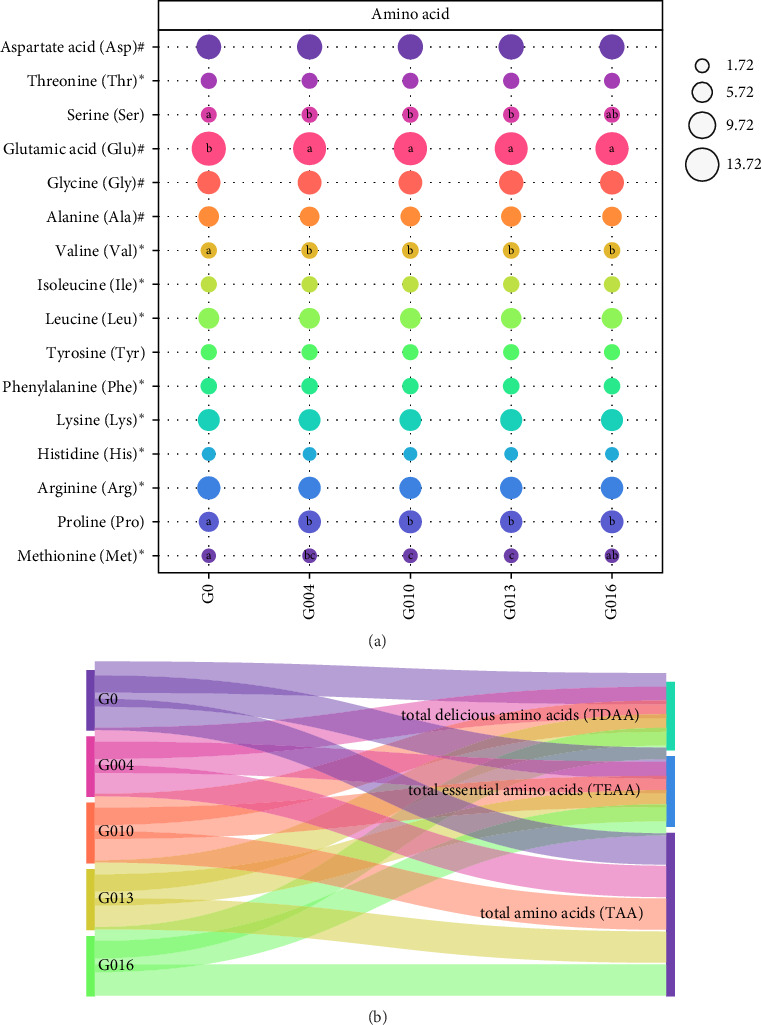
(a, b) Effects of GAA on amino acids in muscle for juvenile *L. vannamei* (on dry matter basis/%, *n* = *3*). Different superscripts in the same row indicate significant differences (*p* < 0.05). *⁣*^*∗*^, essential amino acids; ^#^, nonessential amino acids. GAA, guanidinoacetic acid.

Sixteen amino acids were detected in *L. vannamei* muscle (Figure 3). The contents of serin, valine, proline (*p* < 0.05) and methionine in G004, G010, and G013 groups were all increased significantly, whereas the content of glutamic acid in GAA supplemented groups decreased significantly compared with G0 group (*p* < 0.05). Differences in other amino acids, total essential amino acids (TEAAs), total amino acids (TAAs), and total delicious amino acids (TDAAs) content were not significant (*p* < 0.05).

### 3.3. Antioxidant Index in Serum

The effects of GAA supplements on the antioxidation index are shown in Table 4. SOD activities in serum of G010, G013, and G016 groups were significantly higher than G0 group (*p* = 0.021). With GAA increased, serum MDA levels were all significantly reduced (*p* < 0.001), but no significant differences were found in groups G013 and G016. All groups with added GAA had significantly higher T-AOC than the G0 group (*p* < 0.001), but no significant difference was observed between the G010, G013, and G016 groups.

### 3.4. Immune-Related Enzymes in Hepatopancreas

ACP, AKP, PO, and LZM in hepatopancreas were all first increased and then decreased with GAA increased (Table 5). The activities of AKP (*p* < 0.001) and ACP (*p* < 0.001) in G010 group were significantly higher than other groups. PO activities in G013 group were significantly higher than the control, G004, and G016 groups (*p* < 0.001), but there was no difference with G010 group. LZM activity in G013 group were significantly higher than other groups (*p* = 0.001).

### 3.5. Immune-Related Genes mRNA Expression in Hepatopancreas

**Figure 4:**
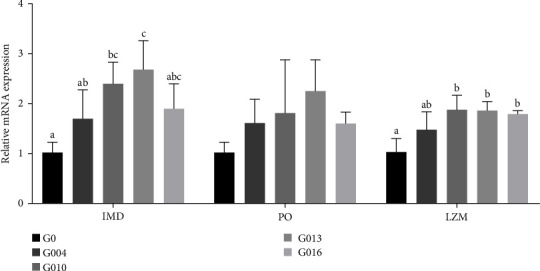
Effect of GAA on immune-related genes mRNA expression in hepatopancreas for juvenile *L. vannamei*. Different superscripts on the columns of the same gene indicated significant differences. GAA, guanidinoacetic acid; *imd*, immune deficiency; *po*, phenoloxidase; *lzm*, lysozyme.

The immune-related gene mRNA expressions in hepatopancreas are presented in Figure 4. The *imd* mRNA expression was significantly upregulated in G010 and G013 groups compared with G0 group (*p* = 0.014), and no significant difference was observed among the other groups. There were no statistical changes of *po* mRNA expression among all the groups (*p* = 0.251). *Lzm* expression in G010, G013, and G016 groups were significantly upregulated than G0 group (*p* = 0.010), and there was no significant difference between G004 group and other groups.

### 3.6. Challenge Test of *Vibrio harveyi*

**Figure 5:**
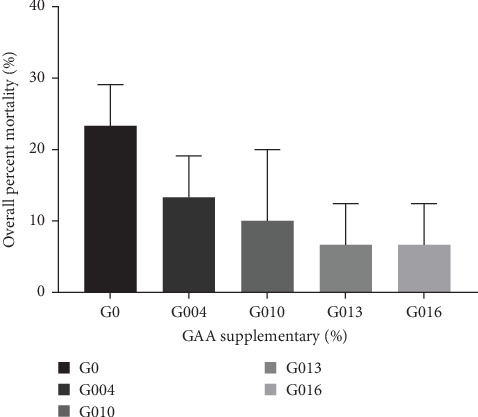
The overall percent mortality of *L. vannamei* at 7 days postchallenge with *V. harveyi* (LD_50_, 2.0 × 10^8^ CFU/mL).

The result of *L. vannamei* challenged by *V. harveyi* is presented in Figure 5. After challenge with *V. harveyi* for 7 days, the overall percent mortality of shrimp showed a gradually decreasing trend with the increase of GAA supplementary but not significantly different from each other (*p* = 0.068). And in all treatment groups, the overall percent mortality in G013 and G016 groups was the lowest (6.67%).

## 4. Discussion

GAA is an endogenous substance for creatine synthesis in human and animals [44]. Numerous studies have shown that GAA can significantly improve the growth performance for aquatic animals [20, 21, 24]. Yang et al. [17] demonstrated that GAA supplementation totally based on vegetable meal diet elevated the growth performance for grass carp (*Ctenopharygodon idella*). In addition, 0.12% GAA was able to significantly increase WGR and SGR and significantly reduce FCR for tilapia [20]. It was reported that the growth-promoting effect of GAA could be attributed to the enhancement of creatine synthesis [45, 46]. Similar findings were obtained in this study. In the present results, 0.10% and 0.13% GAA significantly increased WGR and SGR, that is, GAA is useful in promoting the growth for juvenile shrimp. Meanwhile, 0.13% GAA significantly reduced FCR, indicating that GAA could improve the feed utilization for *L. vannamei* effectively. The aforementioned results showed that GAA could promote the growth and improve the feed utilization of aquatic animals to a certain extent. Therefore, in this study, based on the fold model corresponding to WGR, the appropriate level of GAA addition in dietary feed was 0.10%, significantly improving the growth performance for *L. vannamei*.

The nutritional value of muscle is closely related to essential amino acid (EAA) content, and muscle flavor is related to its composition and content of flavorful amino acids [47]. In non-EAA (NEAA), some amino acids can reflect the umami taste or flavor of muscle, and therefore considered as “delicious” amino acids (DAAs), such as aspartic acid (Asp), glycine (Gly), glutamic acid (Glu), and alanine (Ala). The delicious degree of shrimp mainly depends on the composition and content of DAA in muscle [48]. Glu and Asp are characteristic amino acids that present the umami taste, whereas Gly and Ala are characteristic amino acids that present sweetness [49]. Wiriyapattanasub et al. [21] showed that the increase in dietary GAA content had no effect on the amino acid content in Nile Tilapia. Similar findings were obtained in this study. From the amino acid analysis, it was observed that no significant difference in TAA content was observed among the groups in these results. In addition, GAA levels had a significant effect on muscle content of several amino acids only (Ser, Glu, Val, Pro, Met) but not on TDAA and TEAA content. Therefore, in this study, GAA supplementation in diets did not improve the amino acid content and flavor in shrimp muscle.

Presently, studies suggest that GAA may affect the oxidative-antioxidant system by acting as a superoxide and antioxidant compound [42, 43]. GAA could increase catalase, SOD, and glutathione peroxidase activities in serum and reduce MDA content [50]. SOD is an important antioxidant enzyme in animal body that can remove superoxide root anion effectively [51]. The content of MDA in serum is an important indicator to judge the degree of peroxidation [52]. The studies have shown that GAA in the diets could increase significantly SOD and GSH activities for bullfrog [53] and Nile tilapia [20]. In this study, GAA in diets significantly increased the SOD activity and T-AOC in serum for juvenile *L. vannamei* whereas decreased the content of MDA. These results indicated that GAA dietary supplements could improve AOC of aquatic animals to a certain extent. The antioxidant effect of GAA may be closely related to its ability to increase creatine level in body [46]. Lawler et al. [54] found in cell-free in vitro studies that high creatine concentration could eliminate free radicals. In addition, it was found that phosphocreatine could reduce adriamycin-induced oxidative stress and achieve myocardial protection by decreasing MDA content, increasing the function of antioxidant enzyme systems such as SOD and CAT, and decreasing the effects of adriamycin-induced oxidative stress [55]. Therefore, GAA can, to some extent, improve the AOC for shrimp by increasing antioxidant enzyme activity and thus the AOC.

*L. vannamei*, a very simple aquatic animal, has only a simple digestive system (stomach, hepatopancreas, and intestines) and lacks a complete specific immune mechanism [56]. Thus, *L. vannamei* mainly completes the body's immune response against pathogenic microorganisms through nonspecific immune responses [57, 58]. In particular, the hepatopancreas is the largest immune organ in *L. vannamei*, with detoxification and elimination functions. PO, ACP, and AKP are vital indicators of nonspecific immunity within the hepatopancreas in *L. vannamei* [59]. In general, it is believed that AKP and ACP are involved in the immune defence in aquatic animals and belong to the group on phosphatases capable of hydrolyzing organophospholipids [60]. Meanwhile, AKP directly correlates with crustacean calcium and phosphorus uptake and chitin formation and secretion and is involved in immune system catalysis and metabolism. As for ACP, it is a marker enzyme for organismal macrophage lysosomes and it reflects the degree for macrophage activation [61]. PO is produced by the pro-PO system, removing hydrogen peroxide from the organism, closely related to crustacean immunity [62]. LZM can disrupt the *β*-1,4-glycosidic bond between N-acetylcytidylic acid and N-acetylglucosamine in the bacterial cell wall, thus digesting and decomposing bacteria, inhibiting the growth of exogenous microorganisms, and enhancing immunity. In this study, GAA significantly increased PO, LZM, ACP, and AKP activities in shrimp. Additionally, *imd* encodes a death domain e-containing protein similar to that of receptor interacting protein (RIP) of the tumor necrosis factor receptor (TNF-R) pathway. The *imd* is a key factor in the *imd* signaling pathway to receive intracellular and extracellular signals and initiate signal transduction, then stimulate a series of cascade reactions to form immune influencing factors (such as antimicrobial peptides) [63]. In this study, GAA significantly upregulates *imd* mRNA expression.

For further investigation on the ability of GAA to enhance nonspecific immunity in shrimp, an artificial bacterial infection test was conducted in this study. Artificial bacterial infection test is an effective method to evaluate the nonspecific immunity and disease resistance for shrimp [60, 64]. The study shows that GAA, a positive significance for disease prevention, could enhance the body's immunity [5, 27]. Aziza et al. [20] emphasized that 0.18% GAA significantly downregulated interleukin 1*β* and tumor necrosis factor genes mRNA expression in Nile tilapia whereas upregulating transforming growth factor *β*1 gene expression, thereby indicating the potential role of GAA as an anti-inflammatory agent. In this study, *V. haveyi* was used as the pathogen. After 7 days of observation, the overall mortality of shrimp gradually decreased with GAA increased. It is speculated that creatine (a metabolite of GAA) and its derivatives play a role in enhancing human and animal immunity, especially inflammatory response and cytokine regulation [65, 66]. Therefore, GAA was able to significantly increase the nonspecific immunity for shrimp, thus improving their disease resistance.

## 5. Conclusion

In conclusion, under the conditions in this study, the following conclusions were drawn:

1. 0.10% and 0.13% GAA supplementation in diets significantly increased WGR and SGR and significantly reduced FCR in shrimp.
2. 0.10%, 0.13%, and 0.16% GAA supplementation in diets were effective in increasing SOD and T-AOC activities and significantly reducing MDA in shrimp.
3. 0.10%, 0.13%, and 0.16% GAA supplementation in diets were all effective in increasing LZM, ACP, AKP, and PO activities in shrimp and significantly upregulated IBM and LZM expression.
4. Based on the quadratic regression model of WGR corresponding to GAA supplementary, the optimal supplementary of dietary GAA was 0.10%.

## Figures and Tables

**Table 1:** Formulation and proximate composition of the experimental diets (% dry matter).

Ingredients	Experimental diets				
	G0	G004	G010	G013	G016
Brown fish meal	20.00	20.00	20.00	20.00	20.00
Soybean meal	15.00	15.00	15.00	15.00	15.00
Peanut meal	10.00	10.00	10.00	10.00	10.00
Corn gluten meal	8.00	8.00	8.00	8.00	8.00
Beer yeast	6.00	6.00	6.00	6.00	6.00
Shrimp shell powder	6.00	6.00	6.00	6.00	6.00
Wheat flour	23.00	23.00	23.00	23.00	23.00
Calcium dihydrogen phosphate	1.50	1.50	1.50	1.50	1.50
Vitamin C	0.03	0.03	0.03	0.03	0.03
Choline chloride	0.05	0.05	0.05	0.05	0.05
Phospholipids	1.50	1.50	1.50	1.50	1.50
Soybean oil + fish oil (1 : 1)	2.50	2.50	2.50	2.50	2.50
Mineral premix^a^	0.50	0.50	0.50	0.50	0.50
Vitamin premix^b^	0.20	0.20	0.20	0.20	0.20
GAA	0.00	0.04	0.10	0.13	0.16
Microcrystalline cellulose	5.72	5.68	5.62	5.59	5.56
Total	100.00	100.00	100.00	100.00	100.00
Proximate composition (% dry matter)**^c^**					
Moisture	7.19	7.37	7.52	7.62	7.57
Crude protein	43.87	43.52	43.79	43.68	43.36
Crude lipid	7.62	7.88	7.90	7.88	7.61
Crude ash	10.48	10.38	10.43	10.99	10.65

^a^Contained the following per kg of the mineral premix: KIO_3_ 40.03 g, CoCl_2_ 4.07 g, CuSO_4_ 19.84 g, FeC_6_H_5_O_7_ 13.71 g, ZnSO_4_ 28.28 g, MgSO_4_ 0.12 g, MnSO_4_ 12.43 g, KCl 15.33 g, Na_2_SeO_3_ 2.00 g, and zeolite power 864.19 g.

^b^Contained the following per kg of the vitamin premix: retinyl acetate 10.00 g, VD_3_ 50.00 g, VE 99.00 g, VK 5.00 g, VB_1_ 25.50 g, VB_2_ 25.00 g, VB_6_ 50.00 g, VB_12_ 0.10 g, calcium pantothenate 61.00 g, nicotinic acid 101.00 g, biotin 25.00 g, inositol 153.06 g, folic acid 6.25 g, and cellulose 389.09 g.

^c^Crude protein, crude lipid, crude ash contents, and moisture were measured values.

**Table 2:** The primers used in real-time quantitative PCR.

Genes	Primer sequence (5′-3′)	GenBank
*β-actin*	F: AAGTAGCCGCCCTGGTT	AF300705.2
	R: GATACCTCGCTTGCTCTGG	
*imd*	F: CGGCTCTGCGGTTCACAT	FJ592176.1
	R: CCTCGACCTTGTCTCGTTCCT	
*po*	F: GCCTTGGCAACGCTTTCA	EF115296.1
	R: CGCGCATCAGTTCAGTTTGT	
*lzm*	F: ACTGGTGCGGAAGCGACTA	AF425673.1
	R: GTAAGCCACCCAGGCAGAA	

Abbreviations: F, forward; *imd*, immune deficiency; *lzm*, lysozyme; *po*, phenoloxidase; R, reverse.

**Table 3:** Effects of GAA on growth performance for juvenile *L. vannamei*.

Items	Treatment					*p*-Values
	G0 (Control)	G004	G010	G013	G016	
FBW (g)	8.44 ± 0.07^a^	8.53 ± 0.05^a^	8.72 ± 0.17^b^	8.79 ± 0.04^b^	8.54 ± 0.05^a^	0.030
WGR (%)	3024.54 ± 23.61^a^	3059.82 ± 19.92^a^	3127.92 ± 61.73^b^	3157.29 ± 12.28^b^	3062.44 ± 19.04^a^	0.030
SGR (%/d)	6.15 ± 0.02^a^	6.17 ± 0.01^ab^	6.21 ± 0.04^bc^	6.22 ± 0.01^c^	6.17 ± 0.01^a^	0.040
FCR	1.39 ± 0.02^b^	1.38 ± 0.01^b^	1.36 ± 0.03^ab^	1.34 ± 0.01^a^	1.38 ± 0.01^b^	0.014
SR (%)	95.00 ± 5.00	99.17 ± 1.44	98.33 ± 1.44	97.50 ± 2.50	99.17 ± 1.40	0.371

*Note*: Values in the table are mean ± SD (*n* = *3*). Different superscripts in the same row indicate significant differences (*p* < 0.05).

Abbreviations: FBW, final body weight; FCR, feed conversion rate; GAA, guanidinoacetic acid; SGR, specific growth rate; SR, survival rate; WGR, weight gain rate.

**Table 4:** Effects of GAA on serum antioxidant index for juvenile *L. vannamei*.

Items	Treatment					*p*-Values
	G0 (Control)	G004	G010	G013	G016	
SOD (U/mL)	450.39 ± 33.52^a^	475.59 ± 21.30^ab^	521.26 ± 11.89^b^	518.11 ± 19.09^b^	505.51 ± 28.74^b^	0.021
MDA (nmol/mL)	28.89 ± 0.39^d^	26.92 ± 1.33^c^	24.62 ± 0.92^b^	20.34 ± 1.04^a^	19.57 ± 1.32^a^	<0.001
T-AOC (mM)	0.17 ± 0.00^a^	0.24 ± 0.02^b^	0.30 ± 0.02^c^	0.31 ± 0.02^c^	0.29 ± 0.03^c^	<0.001

*Note*: Values in the table are mean ± SD (*n* = *3*). Different superscripts in the same row indicate significant differences (*p* < 0.05).

Abbreviations: GAA, guanidinoacetic acid; MDA, malondialdehyde; SOD, superoxide dismutase; T-AOC, total antioxidant capacity.

**Table 5:** Effects of GAA on hepatopancreas immune-related enzymes for juvenile *L. vannamei*.

Items	Treatment					*p*-Values
	G0 (control)	G004	G010	G013	G016	
AKP (U/g prot)	78.44 ± 3.01^a^	172.82 ± 3.25^b^	191.36 ± 6.92^c^	175.41 ± 4.56^b^	179.12 ± 1.62^b^	<0.001
ACP (U/g prot)	251.17 ± 10.55^a^	391.54 ± 7.60^c^	433.68 ± 1.69^d^	391.71 ± 8.57^c^	293.97 ± 9.35^b^	<0.001
PO (U/g prot)	0.91 ± 0.06^a^	1.60 ± 0.02^b^	1.89 ± 0.21^cd^	2.00 ± 0.06^d^	1.69 ± 0.15^bc^	<0.001
LZM (U/g prot)	3.87 ± 0.57^a^	4.78 ± 0.37^ab^	5.61 ± 1.06^b^	7.07 ± 0.26^c^	5.46 ± 0.30^b^	0.001

*Note*: Values in the table are mean ± SD (*n* = *3*). Different superscripts in the same row indicate significant differences (*p* < 0.05).

Abbreviations: ACP, acid phosphatase; AKP, alkaline phosphatase; GAA, guanidinoacetic acid; LZM, lysozyme; PO, phenoloxidase.

## Data Availability

The data that support the findings of this study are available on request from the corresponding author. The data are not publicly available due to privacy or ethical restrictions.
